# Edaravone Improves Streptozotocin-Induced Memory Impairment via Alleviation of Behavioral Dysfunction, Oxidative Stress, Inflammation, and Histopathological Parameters

**DOI:** 10.1155/2023/9652513

**Published:** 2023-07-12

**Authors:** Mahdieh Anoush, Soroush Bijani, Fatemeh Moslemifar, Fatemeh Jahanpour, Ali Kalantari-Hesari, Mir-Jamal Hosseini

**Affiliations:** ^1^Zanjan Applied Pharmacology Research Center, Zanjan University of Medical sciences, Zanjan, Iran; ^2^Department of Pharmacology and Toxicology, School of Pharmacy, Zanjan University of Medical Sciences, Zanjan, Iran; ^3^Department of Pathobiology, Faculty of Veterinary Science, Bu-Ali Sina University, Hamedan, Iran

## Abstract

Alzheimer's disease (AD), as the main cause of dementia, has a progressive and neurodegenerative pattern with number of cases increasing over the next decades. Therefore, discovering an effective treatment with the ability to invert memory impairment and pathophysiological events of AD seems to be required. The present study performed to investigate the probable effects of Edaravone (EDV) in AD-like disorder induced by intracerebroventricular streptozotocin (ICV-STZ) administration in mice. This study also compares the two different methods of ICV-STZ in the memory impairment induction. NMRI male mice were administrated with 3 mg/kg of STZ for two times during 48 hours span, and after 24 hours, animals were treated with EDV (5 and 10 mg/kg), Donepezil, and Memantine for 14 days. After behavioral tests regarding memory and cognitive function, animals were sacrificed, and the hippocampi were utilized for further analyses. Our results demonstrated that administration of STZ induced memory impairment in the Morris water maze (MWM) test and decreased the discriminative factor in novel object recognition (NOR). The biochemical output shows a significant decrease in ferric reducing antioxidant power (FRAP) and glutathione (GSH) levels followed by increase in malondialdehyde (MDA) and protein carbonylation (PCO) levels. The output showed no difference between the patterns of AD-like disorder induction. Following our treatment groups, administration of EDV (5 and 10 mg/kg), Donepezil, and Memantine significantly improved memory performance and discriminatory behavior. Aforementioned treatments managed to improve FRAP and GSH content of hippocampus, while significantly attenuating MDA, PCO, and nitric oxide overproduction. In addition, no significant difference has been observed between the effect of 5 and 10 mg/kg EDV application. It was supposed that EDV managed to ameliorate memory dysfunction, discriminatory behavior, oxidative stress, and cellular antioxidant power in a dose-independent pattern in mice.

## 1. Introduction

Alzheimer's disease (AD) is the most common form of dementia that presented itself mainly as senior part of community. Affecting the life of 24 million people worldwide with the number of patients doubling every 20 years [1]. The current cost for management of dementia suffering patients is nearly 1% of global gross domestic product, putting a huge burden on social and medical systems [2]. The main pathological aspects of AD are the imbalance in production and clearance of A*β* peptide, which leads to the degeneration of neural cells, deformation of synapsis, and neuroinflammation all these factors influence the function and integrity of brain [3].

The consequent formation of A*β*42 senile plaques attracts microglial response, which leads to overproduction of pro-inflammatory cytokines [tumor necrosis factor alpha (TNF-*α*), Interferon gamma, and Interleukin-1*β* (IL-1*β*)]. The aforementioned events lead to escalation of oxidative stress in neural tissue. Brain tissue carries low level of reduced glutathione (GSH) and high level of iron, which decreases the potency for free radical scavenging and increases the chance of cellular apoptosis [4]. Mitochondrial dysfunction plays a significant role in pathophysiology of AD at primary states by facilitating A*β* deposition, loss of dendritic branches, and Neurofibrillary tangles formation. The higher level of oxidative stress leads to increase of cytoplasmic mtDNA and cytochrome oxidase as a result of significant reduction of mitochondrial size [5]. There is a link between escalation of oxidative stress and dopamine-related symptoms including cognitive decline [6]. It is observed that the brain regions with high A*β*1–42 accumulation also carry high concentration of peroxided lipids as byproduct of free radical rise. Oxidative stress could alter protein phosphorylation pattern, Mammalian target of rapamycin (mTOR) activation, and glucose metabolism leading to neural autophagy [7].

In case of medical therapy, choices are narrow and many medications for AD belong to Acetylcholinesterase (AChE) inhibitors namely rivastigmine and donepezil (DON), showing promising effect in improving memory performance and cognitive impairment in patients. However, demonstrating minor effect in preventing progressive pattern of AD and probable side effects may prevent patients from continuing treatment [8]. DON has demonstrated neuroprotective potency by various mechanisms including increasing neural protection against A*β*42-induced toxicity, decreasing lactate dehydrogenase release as a marker for neural damage as well as activating phosphatidylinositol 3-kinase/protein kinase B/mTOR pathway [9]. Memantine, another frequent AD medication, acts as antagonizing *N*-methyl-d-aspartate (NMDA) receptors in an uncompetitive manner, and improved patients' performance in Boston Naming Test and the Trail Making Test, while slowed the atrophy of right hippocampus [10]. Memantine also prevents neural excitotoxicity to glutamate response and increased the level of soluble A*β* peptide [11]. Most of the novel hybrid components utilized in latest research act via suppressing AChE, scavenging free radicals, and inverting the chelation of redox-active Cu and Fe [6]. Edaravone (EDV), as the selected medication in this research, has been proved to show significant effects in amyotrophic lateral sclerosis (ALS) treatment in phase III clinical studies. However, the overall mechanism for this action is not broadly discovered, and some studies linked these effects to neuroprotection by scavenging peroxyl water soluble and insoluble radicals and suppressing the inflammatory microglial response in ALS [12, 13].

The main objective of this study was to investigate probable neuroprotective effect of EDV against memory cognitive impairment in mice as a model for AD. A validated intracerebroventricular (ICV) injection of streptozotocin (STZ) was utilized for AD-like pattern induction. Then, memory function and ability were performed by Morris water maze (MWM) test and novel objective recognition (NOR) task. Then, oxidative stress biomarkers and nitric oxide (NO) levels were measured in the brains of AD animal models.

## 2. Materials and Methods

### 2.1. Animals

NMRI male mice (25–30 g) were purchased from Pasteur Institute, Tehran, Iran. Animals were kept under standard laboratory condition including 21–23°C, free access to water/food, and 12 hours of dark/light cycle. All tests on rodents were performed between 10:00 AM and 2:00 PM according to National Institutes of Health guidelines for animal experiments, and approved by the animal ethic committee of Zanjan University of Medical Sciences (Ethical Code: ZUMS.REC.1399.178).

### 2.2. Drugs and Treatments

DON, Memantine, and STZ were dissolved in sterile physiological saline (0.9%), and EDV was dispersed in 1% Dimethyl sulfoxide (DMSO) and physiological saline (0.9%) solution, resulted in a 0.15 mg/ml dispersion. To exclude the probable effects of EDV co-solvent, the control group received the same DMSO and saline solution. Intracerebral STZ injection method for memory impairment was performed using a 2.5 nm syringe injecting 3 mg/kg of STZ in 4 *μ*l solution in left or right cerebrum of the anesthetized mice. The dosage and pattern of injection for STZ were based on Kosaraju et al. [14] study. 24 hours after the last injection, animals were subjected to the behavioral tests including MWM test and NOR (*n* = 7–8). Different set of animals were utilized for biochemical assessments (*n* = 3–4).

### 2.3. Experimental Design

To evaluate whether the number of injections would affect memory performance, 24 mice were divided into two groups: Group 1 received one dose of STZ-ICV injection, and after 24 hours, the memory performance was evaluated in MWM task; Group 2 received a second dose of ICV-STZ 48 hours after the first dose, followed by a memory performance test with a 24-hour interval.

**Figure 1:**
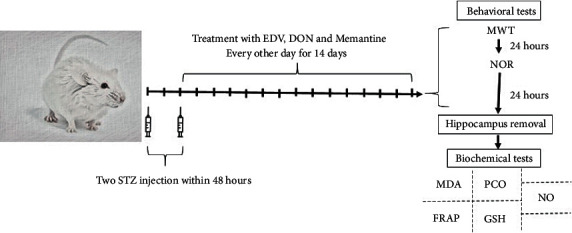
Timeline of ALZ induction procedure, treatment, behavioral, and molecular assessment.

For main research, rodents were randomly divided into nine groups each containing 12 mice (Figure 1). The description of each group is as follows: (1) control group received intraperitoneal vehicle of STZ and/or EDV through ICV and/or intraperitoneal route, respectively, for 14 days. (2) DON group received 1 mg/kg of DON via an IP route every other days for 14 days. (3) Memantine group received 5 mg/kg of Memantine via IP route every other day for 14 days. (4) EDV group received 10 mg/kg of EDV via IP route every other day for 14 days. (5) Alzheimer (ALZ) group received 3 mg/kg STZ via intracerebral route two times in 48 hours interval (after the second method of STZ induction showed less deviation and more sustenance in AD-like disorder symptoms). (6, 7) ALZ + EDV group received 3 mg/kg of ICV-STZ via ICV route with a 48-hour interval; 24 hours later, administration of 5 and 10 mg/kg of EDV started via IP route and repeated every other day for 14 days. (8) ALZ + DON group received 3 mg/kg of ICV-STZ via ICV route in 48 hours interval; 24 hours later, administration of 1 mg/kg DON started via IP route and repeated every other day for 14 days. (9) ALZ + Memantine group received 3 mg/kg of ICV-STZ with a 48-hour interval; 24 hours later, administration of 5 mg/kg Memantine started via IP route and repeated every other day for 14 days. Animals were sacrificed 24 hours after the last behavioral test by cervical dislocation without application of anesthetic, and hippocampi were dissected and frozen at −80 to minimize alterations for biochemical and histopathological assay.

### 2.4. MWM Test

In order to evaluate the effects of intervention on spatial learning and memory function, MWM test was performed. A day after the last injection, animals were submerged in a circular pool kept at 22–25°C. Above the pool, a camera, which was connected to a computer, was settled. All the data about swimming pattern, route, and speed of the animals were recorded by Mazeruter software. Near the surface of water, an escape platform was placed in the presumed Q2 quarter of the round pool. On probe trials, mice were allowed to swim freely in the pool with the escape platform inside, for 60 seconds and stay for 10 seconds on the platform. In case of rodents that were unable to find platform, they were manually placed on the platform for 10 seconds. Training pattern for rodents consisted of four probe trials in 4 consecutive days. A day after the last trial, all animals were placed separately on water maze for 60 seconds, while the escape platform had been removed. The normalized time animals spent on Q2 zone of the maze (former place of the escape platform) was calculated for each rodent by El-Sahar et al. [15].

### 2.5. Novel Object Recognition

Cognitive impairment is one of the noticeable aspects of AD, in order to assess the tendency of rodents to spend more time with unfamiliar objects NOR as function of episodic memory, and this test was performed in a box made of Plexiglas (70 × 70 × 30 cm). On first phase, animals were placed in the empty box for 15 minutes each day for 2 days prior to test in order to habituate to unfamiliar environment. After 24 hours on second phase, rodents were placed in the box again to explore similar shaped and sized object placed at equal distance from the center for 10 minutes. On the next day and third phase, animals brought to box found one object is replaced with novel object with different color and shape; rodents left to explore both objects for 3 minutes. Discrimination ratio was calculated by dividing the time rodent spent to explore the novel object by the total time of exploring novel object and familiar ones [DR = (T Novel)/(T Novel + T Familiar)] [16].

### 2.6. Ferric Reducing Antioxidant Power

Ferric reducing antioxidant power (FRAP) assay was utilized to evaluate the total antioxidant potency of the samples in iron(iii) reduction to Iron(ii), in reaction with 2,4,6-Tri(2-pyridyl)-s-triazine (TPTZ) as the colorimetric reagent. The absorbance of the samples was measured at 593 nm [17].

### 2.7. Evaluation of Lipid Peroxidation

Malondialdehyde (MDA) is the main byproduct of the escalation of oxidative stress. In order to measure the scale of lipid peroxidation (LPO) production, the absorbance of MDA with thiobarbituric acid reactive substances (TBARs) was recorded at 532 nm [18, 19].

### 2.8. Evaluation of Reduced GSH

GSH is one of the main endogenous antioxidants in neural tissue, showing a considerable decline of GSH is known as the first marker for the occurrence of neuroinflammation. DTNB [5,5'-Dithiobis (2-nitrobenzoic acid)] was used as the reagent for GSH measurement, and the absorbance was measured at 412 nm [19].

### 2.9. Evaluation of the Protein Carbonylation

Protein carbonylation (PCO) counts as another byproduct in oxidative stress increasing pattern. This measurement was based on PCO reaction with 2,4-dinitrophenylhydrazine, and the absorbance was measured at 365 nm [17].

### 2.10. NO Level Assay

The amount of NO in the brain samples was measured based on spectrophotometric method at 540 nm wavelength following reaction of nitrites with Griess reagent and form purple azo using Zistfanteb commercial kit (Cib Biotech, Iran).

### 2.11. Histopathological Assay

The collected samples were fixed using 4% formalin, and dehydration was performed using 70% ethanol solution. The tissues then were irrigated by xylene and fixed in paraffin block. Finally, 15 *μ*m thickness slices were cut and dyed using hematoxylin and eosin (H&E staining). The prepared slides were examined under Carl Zeiss Axio light microscope, and images were taken for further analyses [20].

### 2.12. Statistical Analysis

Results were expressed as mean ± SD, and R studio programming software was used for statistical analyses. Comparison between the groups was carried out using two-way analysis of variance (ANOVA). Furthermore, to evaluate the main impact of disease induction and treatment as well as their interaction, post hoc Tukey's Honestly significant difference test was applied. *P* < 0.05 was considered statistically significant. Plots were generated using Matplotlib library in Python.

## 3. Results

### 3.1. Effects of Number of STZ Admirations on Water Maze Performance

**Figure 2:**
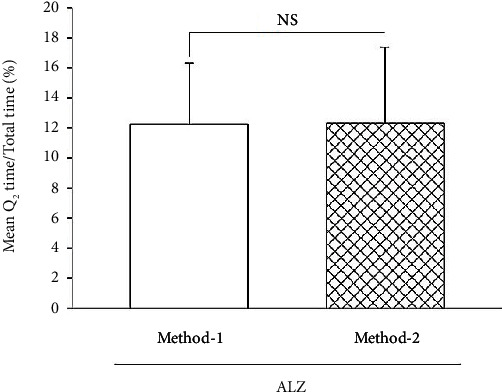
Comparison of the effect of two methods of ALZ-like disorder induction in Morris water maze task (*n* = 12).

As demonstrated in Figure 2, the output of T test analyses shows no significant difference between one administrated dose of STZ and two administrated doses of STZ on induction of memory impairment in mice (*P* > 0.05). Although there is no significant difference between responses in receiving ICV-STZ double injection in 48-hour interval and once prior to behavioral tests, low standard deviation and higher duration time of AD induction following two injections of ICV-STZ were more relative model for AD induction than one injection of ICV-STZ.

### 3.2. Effects of ALZ Induction and Treatment on Water Maze Task Performance

**Figure 3:**
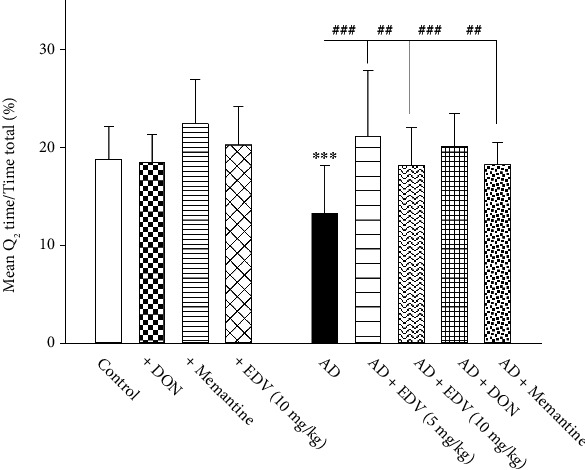
Effects of ALZ induction and different treatments on rodents' performance in water maze task. Values are expressed as the mean ± SD and were analyzed using one-way ANOVA followed by Tukey's post hoc test. Significant difference was accepted at (∗#*p* < 0.05, ∗∗##*p* < 0.01, ∗∗∗###*p* < 0.001) (*n* = 7 − 8).

Two-way ANOVA analysis showed significant effects of ALZ induction [*F* = 1.031; *P* < 0.314], treatment [*F* = 4; *P* = 0.006], and ALZ induction × treatment interaction [*F* = 1.569; *P* = 0.192] on MWM test. Results of Figure 3 express that ICV-STZ caused memory impairment in mice by decreasing their presence in Q2 zone significantly when compared to control group (*P* < 0.001) by 37.5%. Treatment with Memantine, DON, and EDV for 14 days has considerably improved animal performance in MWM in comparison to ALZ group (*P* < 0.01) by 52% for ALZ + Memantine and ALZ + EDV 10 groups, 60% for ALZ + EDV 5, and 76% for ALZ + DON, respectively. The differences in the memory improvement levels between 5 and 10 mg/kg doses of EDV were insignificant (*P* > 0.05). DON, Memantine, and EDV did not induce any alteration on memory performance in intact mice (Figure 3; *P* > 0.05).

### 3.3. Effects of ALZ Induction and Treatment on NOR Performance

**Figure 4:**
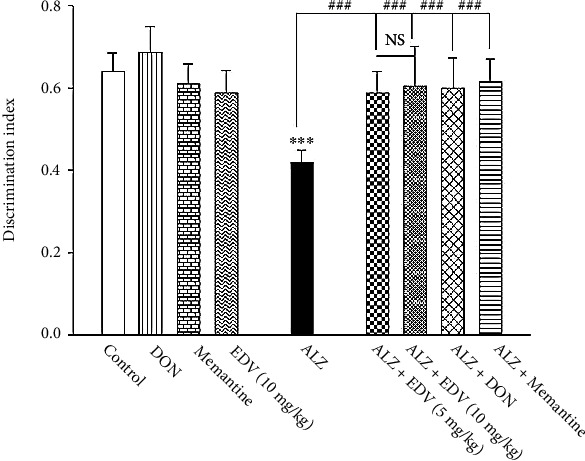
Effects of ALZ induction and different treatments on rodents' performance in novel object recognition. Values are expressed as the mean ± SD and were analyzed using one-way ANOVA followed by Tukey's post hoc test. Significant difference was accepted at (∗#*p* < 0.05, ∗∗##*p* < 0.01, ∗∗∗###*p* < 0.001) (*n* = 7 − 8).

Two-way ANOVA analysis showed significant effects of ALZ induction [*F* = 7.86; *P* = 0.006], treatment [*F* = 6.585; *P* < 0.001], and interaction [*F* = 4.733; *P* = 0.002] on NOR performance. As presented in Figure 4, STZ administration considerably decreased animal tendency for novel objects (*P* < 0.001) by 34%. As for 14 days of treatment with Memantine, DON, and EDV, all medication managed to improve rodent's tendency to novel objects while compared to ALZ group (*P* < 0.001) by 40–42% in all treatment groups. DON, Memantine, and EDV groups did not demonstrate any significant alteration in discrimination factor when compared to control group (Figure 4; *P* > 0.05).

### 3.4. Effects of ALZ Induction and Treatment on FRAP Level

**Figure 5:**
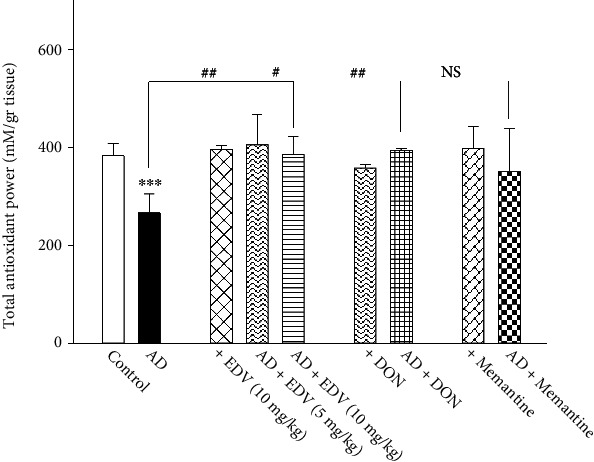
Effects of ALZ induction and different treatments on the hippocampal amount of FRAP. Values are expressed as the mean ± SD and were analyzed using one-way ANOVA followed by Tukey's post hoc test. Significant difference was accepted at (∗#*p* < 0.05, ∗∗##*p* < 0.01, ∗∗∗###*p* < 0.001) (*n* = 3 − 4).

Two-way ANOVA analysis showed significant effects of ALZ induction [*F* = 3884; *P* < 0.059], treatment [*F* = 4.201; *P* = 0.009], and ALZ induction × treatment interaction [*F* = 3.677; *P* = 0.017] on FRAP level. As the data in Figure 5 depicts, STZ administration significantly declined total cellular antioxidant level compared to control group (*P* < 0.001) by 44%. Treatment with Memantine, DON, and EDV has significantly improved cellular FRAP level in comparison to ALZ group (Figure 5; *P* < 0.001) by 72–80%. The FRAP recovery potency of EDV between two dosage groups was insignificant (*P* > 0.05). The aforementioned medications did not alter FRAP level when administered to normal animals (*P* > 0.05).

### 3.5. Effects of ALZ Induction and Treatment on GSH Level

**Figure 6:**
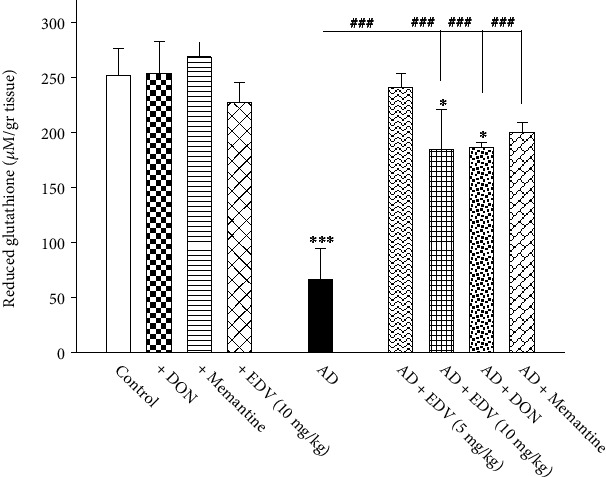
Effects of ALZ induction and different treatments on the hippocampal amount of GSH. Values are expressed as the mean ± SD and were analyzed using one-way ANOVA followed by Tukey's post hoc test. Significant difference was accepted at (∗#*p* < 0.05, ∗∗##*p* < 0.01, ∗∗∗###*p* < 0.001) (*n* = 3 − 4).

Two-way ANOVA analysis showed significant effects of ALZ induction [*F* = 80.857; *P* < 0.001], treatment [*F* = 13.368; *P* = 0.001], and ALZ induction × treatment interaction [*F* = 17.84; *P* = 0.001] on GSH level. The illustrated Figure 6 indicates that ALZ group shows significantly lower amount for GSH compared to control group (*P* < 0.001) by 53%. Memantine, DON, and EDV treatments managed to recover GSH resources in comparison with ALZ group (Figure 6; *P* < 0.001) by 107–123%. These three interventions did not alter the GSH levels of brain when applied to normal mice (*P* > 0.05).

### 3.6. Effects of ALZ Induction and Treatment on MDA Level

**Figure 7:**
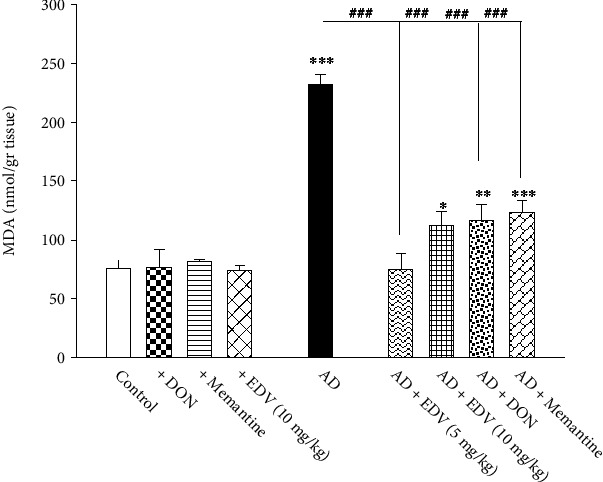
Effects of ALZ induction and different treatments on the hippocampal amount of MDA. Values are expressed as the mean ± SD and were analyzed using one-way ANOVA followed by Tukey's post hoc test. Significant difference was accepted at (∗#*p* < 0.05, ∗∗##*p* < 0.01, ∗∗∗###*p* < 0.001) (*n* = 3 − 4).

Two-way ANOVA analysis showed significant effects of ALZ induction [*F* = 248.112; *P* < 0.001], treatment [*F* = 55.489; *P* = 0.001], and ALZ induction × treatment interaction [*F* = 54.289; *P* = 0.001] on MDA level.  Figure 7 reveals that ICV-STZ administration escalates MDA production in ALZ group compared to control group by 380%. In addition, treatment of ICV-STZ with Memantine, DON, and EDV significantly dropped the overproduction of MDA after 14 days of therapy compared to ALZ group (Figure 7; *P* < 0.001) by 70–79%. Concerning the effect of all selected treatments on healthy animals, neither of them made significant alteration in MDA compared to control group.

### 3.7. Effects of ALZ Induction and Treatment on PCO Levels

**Figure 8:**
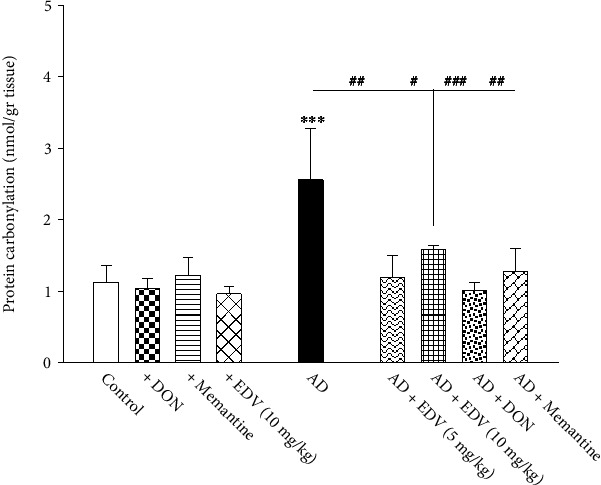
Effects of ALZ induction and different treatments on the hippocampal amount of PCO. Values are expressed as the mean ± SD and were analyzed using one-way ANOVA followed by Tukey's post hoc test. Significant difference was accepted at (∗#*p* < 0.05, ∗∗##*p* < 0.01, ∗∗∗###*p* < 0.001) (*n* = 3 − 4).

Two-way ANOVA analysis showed significant effects of ALZ induction [*F* = 18.01; *P* < 0.001], treatment [*F* = 8.796; *P* = 0.001], and ALZ induction × treatment interaction [*F* = 7.364; *P* = 0.001] on PCO level. As presented in Figure 8, ALZ group demonstrated a significantly higher amount for PCO compared to control group (Figure 8; *P* < 0.001) by 75%. Memantine, DON, and EDV managed to recover overproduction of PCO significantly compared to ALZ group (*P* < 0.001) by 42–45%.

### 3.8. Effects of ALZ Induction and Treatment on NO Levels

**Figure 9:**
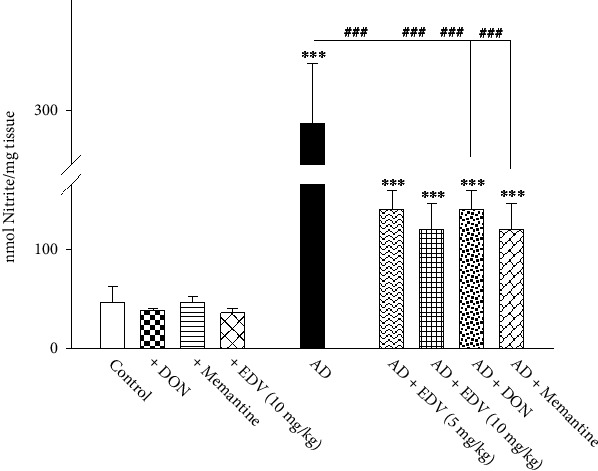
Effects of ALZ induction and different treatments on the hippocampal amount of nitric oxide. Values are expressed as the mean ± SD and were analyzed using one-way ANOVA followed by Tukey's post hoc test. Significant difference was accepted at (∗#*p* < 0.05, ∗∗##*p* < 0.01, ∗∗∗###*p* < 0.001) (*n* = 4).

Two-way ANOVA analysis showed significant effects of ALZ induction [*F* = 211.18; *P* < 0.001], treatment [*F* = 9.638; *P* = 0.001], and ALZ induction × treatment interaction [*F* = 10.184; *P* = 0.001] on NO level. As demonstrated in Figure 9, ICV-STZ caused significant surge in NO levels compared to control group (532% increase, *P* < 0.001). Further treatment with EDV (5 and 10 mg/kg), DON, and memantine has noticeably dropped the surge of NO levels compared to ALZ group (52%, 59%, 34%, and 57%, respectively, *P* < 0.001). However, NO levels in three aforementioned groups were yet significantly higher than that of control group (Figure 9; *P* < 0.001). The administration of DON, Memantine, and EDV shows no significant changes in NO percentage amounts.

### 3.9. The Effect of Intervention on Histopathological Output

**Figure 10:**
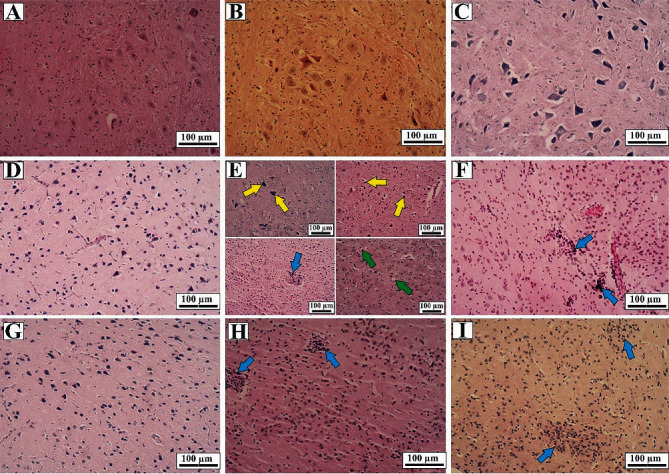
Hippocampus sections in control and treatments groups (*n* = 3). H&E staining. ×100. (a) Control group received of STZ and/or EDV; (b) DON group received 1 mg/kg of Donepezil; (c) Memantine group received 5 mg/kg of Memantine; (d) EDV group received 10 mg/kg of EDV; (e) ALZ group received 3 mg/kg, which received ICV-STZ two times in 48 hours interval and receiving ICV-STZ once: basophilic necrotic neuron (yellow arrows), microglial nodule (blue arrow), and mild vacuolization (green arrows); (f) ALZ + EDV group received 3 mg/kg of ICV-STZ in 48 hours interval; 24 hours later administered with 5 mg/kg of EDV: microglial nodule (blue arrows); (g) ALZ + EDV group received 3 mg/kg of ICV-STZ in 48 hours interval; 24 hours later administered with 10 mg/kg of EDV: there was no damage; (h) ALZ + DON groups received 3 mg/kg of ICV-STZ+1 mg/kg of Donepezil: microglial nodule (blue arrows); (i) ALZ + Memantine group received 3 mg/kg of ICV-STZ + Memantine group received 5 mg/kg of Memantine: microglial nodule (blue arrows).

As in Figure 10 and Table 1, the ICV-STZ results in moderate to severe alteration in basophilic necrotic neuron, vacuolization, and microglial nodule in ALZ group (Figure 9(e)) based on scoring of histopathological changes, while mild microglial nodule was observed in ALZ + EDV (5 mg/kg), ALZ + DON (1 mg/kg), and ALZ + Memantine (5 mg/kg) groups (grade 1) (Figures 10(f), 10(h), and 10(i)) and Table 1). On ALZ + EDV (10 mg/kg), however, no alternation was noted compared to control group (Figure 10(g) and Table 1). The DON, Memantine, and EDV groups demonstrated no histopathological alteration compared to control group (Figures 10(a), 10(b), 10(c), and 10(d) and Table 1).

## 4. Discussion

Alzheimer is one of the main causes of dementia affecting the life of millions of elder communities around the world. While the pathophysiology of AD is widely investigated, the medical approaches in controlling its progression are highly limited. AChEIs are considered the main vastly utilized medication. However, lack of improvement in some cases and sustained reversal of symptoms over time [21] have motivated researchers to investigate novel compounds for this disease.

This study utilized STZ to induce AD-like disorder in mice, and in the very first step compared the effect of two methods of STZ-ICV injections in memory impairment induction (1-single injection and 2-double injection with a 48-hour interval). The results of MWM demonstrated no significant difference between these two methods in memory performance. However, the double injection method resulted in smaller SD amounts and on the other hand, increased the time in which animals demonstrated memory deprivation and other Alzheimer's symptoms. Therefore, the double injection method with a 48-hour interval was applied alongside the project. ICV-STZ in similar studies increased accumulation of A*β*, rapid influx of Ca^2+^ into neural cells, induced mitochondrial dysfunction as well as hyper stimulation of glutamate receptors [22, 23]. ICV-STZ method considerably mimics the phenotype of sporadic AD-like signs as result of insulin resistant brain state [24].

In this model for AD-like disorder, DON and Memantine have been selected as our standard treatments because they have been proved to ameliorate memory and behavior impairments in AD-like rodent models via MWM and NOR tests [25, 26]. In addition, they mitigated byproducts of Reactive Oxygen Species accumulation namely MDA and PCO and improved neural antioxidant protection by recovering total antioxidant amount in FRAP and GSH levels [27]. These effects are not simply due to their direct free radical scavenging properties, but due to their amending effects on synaptic performance and modulating neural cells homeostasis [28]. As for Memantine, which improves GSH production in astrocytes; ameliorates GSH levels, which in turn results in glutamate's higher affinity to NMDA, increasing NMDA channels' activity [29].

In this study, EDV administration managed to improve animals' memory performance in MWM and the discrimination factor in NOR significantly in comparison to AD-like disorder group. In this context, it has been proved that enhancement in the function of peripheral cortex and hippocampus is related to higher rodents' discriminative behavior in NOR [30]. EDV treatment also demonstrated recovering effect on abnormalities induced by ICV-STZ application, namely regulatory effect on FRAP as a representative of total antioxidant resources and GSH as the main endogenous neural antioxidant. In addition, this treatment managed to deprive oxidative stress in neural tissue significantly, via a considerable decline in MDA and PCO further products.

In a relative study, EDV managed to reverse neuroinflammation by modulation of IL-1*β* and NLRP-3 as the key roles of immune response and suppressed neural apoptosis by decreasing the expression of Caspase 1 and NF-*κ*B [31]. EDV, as a lipophilic antioxidant, has managed to increase superoxide dismutase and catalase levels leading to further degradation of hydrogen peroxide and recovery of enzymatic antioxidant protection, which would in turn improve mitochondrial function as the main source of superoxide free radicals [32, 33]. It has been proved that ICV-STZ mainly affects brain signal transduction by increasing A*β* accumulation, MDA formation, TNF-*α*, and IL-6 level, which can be altered by EDV, so that the hippocampal antioxidant resources would be retrieved [34, 35].

In the present study, EDV managed to prevent oxidative stress by declining PCO and MDA products in a dose-independent pattern. No significant difference has been observed between 5 and 10 mg/kg EDV treated mice in controlling oxidative stress. In a relative study, EDV treatment in ICV-STZ rats managed to attenuate MDA levels and recover GSH levels in brain hippocampus, while also retrieving NO levels and AChE activity leading to restoration of blood flow as well as memory impairment [36].

The excessive amount of NO production can mitigate oxidative stress and facilitate the neuroinflammation process by increasing IL-1*β*, IL-6, and TNF-*α* expression and suppressing Brain-derived neurotrophic factor and triggering receptor expressed on myeloid cells 1 (TREM1) [37]. In our study, EDV treatment has deprived the overproduction of NO, which resulted in histopathological abnormalities that consist of microglial nodule formation and vacuolization in Alzheimer-like disorder. This process indicates neural inflammation and necrosis in the similar manner of AD [38]. In conclusion, our findings demonstrate that EDV is capable of attenuating memory impairment, discriminatory behavior, oxidative stress, NO overproduction, and cellular antioxidant power in a dose-independent pattern in mice.

## Figures and Tables

**Table 1:** Grading of histopathological changes in the hippocampus of mice.

Groups	Alteration in basophilic necrotic neuron	Alteration in vacuolization	Alteration in microglial nodule
Control	−	−	−
DON	−	−	−
Memantine	−	−	−
EDV(10 mg/kg)	−	−	−
ALZ	**+**	**++**	**++**
ALZ + EDV(5 mg/kg)	−	−	**+**
ALZ + EDV(10 mg/kg)	−	−	−
ALZ + DON	−	−	+
ALZ + Memantine	−	−	+

Scoring was done as follows: NO (−); Mild (+); Moderate (++); and Severe (+++).

## Data Availability

The data used to support the findings of this study are available from the corresponding author upon request.

## Abbreviations

AD: Alzheimer's disease
ICV: Intracerebroventricular
STZ: Streptozotocin
MWM: Morris water maze
NOR: Novel object recognition
FRAP: Ferric reducing antioxidant power
GSH: Glutathione
MDA: Malondialdehyde
PCO: Protein carbonylation
TNF-*α*: tumor necrosis factor alpha
IFN-*γ*: Interferon gamma
IL-1*α*: Interleukin-1*α*.
